# The Ethics of Error in Medicine

**DOI:** 10.5041/RMMJ.10406

**Published:** 2020-10-14

**Authors:** Shamai A. Grossman, Kiersten L. Gurley, Richard E. Wolfe

**Affiliations:** Department of Emergency Medicine, Beth Israel Deaconess Medical Center, Harvard Medical School, Boston, MA, USA

**Keywords:** Emergency, error, ethics

## Abstract

The practice of medicine forces medical practitioners to make difficult and challenging choices on a daily basis. On the one hand we are obligated to cure with every resource available, while on the other hand we put the patient at risk because our treatments are flawed. To understand the ethics of error in medicine, its moral value, and the effects, error must first be defined. However, definition of error remains elusive, and its incidence has been extraordinarily difficult to quantify. Yet, a health care system that acknowledges error as a consequence of normative ethical practice must create systems to minimize error. Error reduction, in turn, should attempt to decrease patient harm and improve the entire health care system. We discuss a number of ethical and moral considerations that arise from practicing medicine despite anticipated error.

## INTRODUCTION

Throughout history, the practice of medicine has presented practitioners with challenging and difficult decisions. They have been asked to provide healing using every possible resource, while often being forced to place their patients at risk due to flawed treatments, imperfect science, limited resources, financial constraints, and more.

The frequently quoted study by the United States (US) Institute of Medicine (IOM), *To Err is Human*, noted that errors cause between 44,000 and 98,000 deaths every year and over one million injuries.^1^ More recently, error was reported as the third leading cause of death in the US with 250,000 deaths per year accounting for 9.5% of all deaths.^2^ In 2005, medical mistakes, medication errors, or test errors were reported in as many as 34% of all patients in the US—the highest rate of any nation.^3^ A follow-up study in 2016, found little improvement.^4^ Although these early studies classified adverse events as 100% preventable, subsequent reviewers estimate 3%–5% of deaths were probably preventable.^5^

The ethics of error in medicine can only be understood after defining its moral value and its effects. Furthermore, defining error remains elusive, and its incidence, for example, in emergency medicine, is extraordinarily difficult to quantify. In theory, the rate of error in emergency medicine should be extraordinarily high, as the gatekeepers of a multifaceted complex health care system are often the first stop in evaluation and treatment.^6^ Short evaluation time, a hectic environment, minimal or often disjointed and difficult-to-access health care records, and little time for patient–physician rapport-building and shared decision-making may all lead to error in emergency medicine. Yet, one study using automated select case reviews reported an error rate of 9.5%, representing an error rate per year of only 0.13% if the analysis was extended to all emergency department patients.^7^ This discrepancy in error rates is likely multifactorial and affects all medical specialties; physicians and caregivers may be reluctant to report error due to feelings of guilt, fear of retribution from patients, and apprehension related to possible loss of employment or damage to reputation.^7^

In truth, we believe error is subjective. The Merriam–Webster dictionary defines “error” as an act or condition of ignorant or imprudent deviation from a code of behavior.^8^ In the case of medical error, the relevant code of behavior is the standard of care; however, this standard may vary from specialty to specialty and even from institution to institution, compromising our ability to identify deviation from the standard of care. On the other hand, relying on individual judgment without rules or defined standards reinforce subjectivity and high variability in care. Prior data have suggested a lack of inter-rater reliability between case reviewers in assigning error, which may reflect the difficulty distinguishing between judgment calls and errors, as well as varying individual reviewers’ ability to overcome outcome bias in assessing whether an error has been made as well as who made the error.^7^ Furthermore, providers may have distinct thresholds for assigning errors when the desire to protect a colleague conflicts with the duty to improve the system. Studies that have assessed error have often failed to use providers with actual expertise in the standard of care of the particular discipline.^9^ Reviews by providers unfamiliar with a given specialty or local conditions may add to the subjectivity of error assignment. The lack of scientific methodology in error assignment has been identified as a concern as early as 1996 but has rarely been considered even in the studies that supported the opinions in the IOM report.^10^

Even if error is accepted within the ethical practice of medicine, the cost of practicing medicine in a system replete with error may be prohibitive. With health expenses at a critical level, as a society, we cannot afford to allow errors to go undetected. In 1999 the IOM estimated that the annual cost of medical errors ranged from US$17 billion to US$29 billion.^1^ This staggering amount may underestimate the true cost of errors. Yet, errors such as omissions and delays are hard to identify unless they cause serious adverse reportable events. Error may create additional indirect costs, which arise from defensive medical practice and administrative overheads to prevent lawsuits resulting from errors and preventable adverse events. Thus, a health care system that acknowledges error as a consequence of normative ethical practice must create systems to minimize error.

Error reduction, in turn, should attempt to decrease patient harm and improve the entire health care system by shifting resources away from unnecessary care needed to ameliorate the effects of the error, back to helping the patient and caregivers more directly. Such an approach would serve as a model to increase the cost-effectiveness of the health care system and the value of the care delivered.

Despite the complexities in quantifying and defining error and the continuing needs to better refine what constitutes error, error in medicine exists. In reality, we practice medicine based on Bayesian theorem, relying on probabilities to make diagnoses, based on prior knowledge or history of conditions that might be related to the diagnosis and then ordering tests to help confirm or rule out that diagnosis. One would hope that every time we evaluate a patient and test a diagnosis, the post-test probability of that patient having a certain disease will be higher than the pretest probability of their having that disease. Yet, even if we always ordered the correct test, testing is confounded by false positive and false negative results, and additionally, we make diagnoses based on likelihood ratios of a patient having a certain disease, rather than certainty, setting practitioners up for error.

We would like to discuss a number of ethical and moral considerations that arise from practicing medicine in the shadow of error. First, when a practitioner does err, at what point does that practitioner no longer have a right to continue to practice? Second, if the error is egregious such that its outcomes result in loss of life or limb, should this further preclude the practitioner from practicing? Third, should a student, trainee, or junior physician be allowed to train with the assumption that their care will likely be more error-prone then that of the more seasoned physician? Lastly, does an apology ameliorate error, or is it futile?

Our response to each of these four ethical concerns is discussed below.

## FIRST ETHICAL CONCERN: PRACTICING MEDICINE AFTER AN ERROR

### At what point does a practitioner no longer have a right to continue to practice?

Assuming a pool of infinite doctors, one might argue that one should withdraw from medical practice following an error. However, if physicians knew they would be forced to stop practice after an error, few physicians would even enter practice. For those who choose to acknowledge the risk of error in the practice of medicine, their clinical practice may also be paralyzed, becoming far too conservative in their management decisions, potentially resulting in excess costs, resource overutilization, and harm caused by overdiagnosis or overtreatment. In addition, not all errors are equal in magnitude or in consequences. As decisions by caregivers are made in real time, each decision has a risk/benefit ratio that must balance multiple potential etiologies and outcomes. Every practitioner is at risk of making decisions that can cause harm such as through lack of knowledge, lack of attention, or lack of judgment. Even with the best intent of helping the patient, errors will almost certainly occur for all physicians at some point in time.

Tosefta, a compilation of Jewish oral law from the late 2nd century, notes that when an expert physician receives licensing from the courts to practice medicine and subsequently harms a patient through negligence, he is exempt from payment due to societal need.^11^ Society needs physicians. Ceasing to practice would shorten life spans and allow illness to proliferate exponentially.

We often assume that a physician will be cognizant of an error. Yet in practice they may be unaware. Society has accepted that the practice of medicine is imperfect. Despite expectations of healing, society nevertheless acknowledges that its members will err in their attempts to find and implement treatment. Similarly, physicians acknowledge that they will make mistakes and even inflict harm in their attempts to ameliorate disease. Humanity is obligated to take some risk in order to preserve human life.

Clearly practitioners must be exceedingly cautious to avoid error. Nevertheless, in order to practice medicine, we believe we are first bound to acknowledge that we will err and when we do err that we must double our commitment to avoid future error. Without a doubt there are patients who could be better treated and procedures that could be better performed by other practitioners. However, if one has accumulated the requisite qualifications to care for a patient or perform procedure, one should be allowed to care for the patient, in the knowledge that all physicians err.

## SECOND ETHICAL CONCERN: EGREGIOUS ERROR

### Should egregious error, such that its outcomes result in loss of life or limb, categorically preclude the physician from practicing medicine?

There must be some sense of accountability. In the US there is a system of checks and balances. Public reporting of error and adverse events to the Department of Public Health and Board of Medicine, although designed to minimize recurrence via increased awareness and development of systems for prevention, by definition elicits deterrence. Similarly, one would expect the more one is engaged in litigation, the greater the pressure to avoid error. Conversely, if medicine becomes less regulated and physician concern for malpractice more limited, physicians may be become more cavalier in their care and error may subsequently increase.

Once a physician is aware that the risks of medical intervention may add no significant benefit when compared to allowing the disease to take its natural course, continuing to provide care for reasons of income or prestige may be unethical. Our profession requires peer regulation, not self-regulation which requires a self-awareness and self-control that is commonly absent in many cases of problem providers.

Prior data suggest that following an egregious error, physicians foster higher levels of anxiety regarding future errors, as well as suffering loss of confidence, sleeping difficulties, reduced job satisfaction, and harm to their reputation. Anxiety is particularly high when they fear litigation. Even with less serious errors, one-third of physicians note increased stress.^12^

Public reporting and peer assessment ideally enable assessment of the advisability or permissibility of future practice following an egregious error, such as one that resulted in loss of life or limb. In the less ideal world, these physicians continue to practice, at times self-regulating based on their own perception or anxieties reflecting a need to change their practice following an error. At times error may be the result of substance abuse, or the stresses of error may foster addiction. Identification and regulation of addiction must be ingrained as part of health care on both departmental and institutional levels.

An analysis of 2,974 malpractice claims in Canada found that most were related to some form of physician error.^13^ In the United States, medical error is usually related specifically to the treatment itself, medication errors, incorrect testing results, delay in notification about abnormal tests, and lapses in communication during transitions between health care providers.^12^ Germany reports a high error rate due to lack of follow-up care.^12^

The United States has been heavily criticized for its malpractice system where, given the large rewards and the difficulty proving negligence, there is limited incentive for health care providers to reveal details about what occurred, or even report errors that might lead to prevention. In contrast, Denmark as well the other Scandinavian countries and New Zealand, rather than compensating based on individual malpractice claims, compensate patients systematically for error and are thus more readily able to gather data from claims by identifying providers and then use this data to ameliorate future events.^14^ This argues for a less litigious and less malpractice-oriented health care system.

In reality all errors are different and few are egregious. As noted above, early studies looked at adverse events as 100% preventable, thus all subject to error, yet subsequent reviewers substantially disproved this.^5^ In fact, there are adverse events even when everything was done properly; these are not errors. Then, there are errors of judgment related to human imperfection in decision-making. Similarly, there are errors with devastating outcomes due to minor deviations from accepted norms of practice or due to knowledge gaps characteristic of the training of young physicians. Some errors are related to faulty systems or technological failures, beyond the control of the practitioner. Real-world practice demands a different response for each type of error regardless of severity of outcome.

## THIRD ETHICAL CONCERN: THE INEXPERIENCED PHYSICIAN

### Should a trainee or junior physician be allowed to train with the assumption that their care will likely be more error-prone than that of the more seasoned physician?

On the one hand, depending on circumstance, the assumption that inexperienced physicians are more error-prone may be valid. However, if trainees never care for patients or perform procedures then they will never stop being trainees, nor accumulate adequate expertise to work as a senior practitioner. On the other hand, care from a junior practitioner—infused by youthful vigor, intellectual curiosity, and more recent education—might surpass that of a senior practitioner. Several recent studies of high-risk cardiac patients hospitalized in teaching hospitals had a lower 30-day mortality when admitted during national cardiology meetings when much of the senior faculty was away, suggesting that care by less experienced or less specialized providers can actually improve medical outcomes.^15^,^16^ Along these lines, a recent study found that seasoned physicians who on survey revealed signs of burnout were 2.2 times as likely to report a medical error.^13^

A junior physician, nevertheless, may not perform a procedure without adequate expertise unless appropriately supervised. Additionally, patients retain the right to refuse care from a trainee and may demand care from a supervising physician. In the setting of physician shortages, a trainee who by definition has a greater likelihood of error, may be the only source of medical care.

A growing literature suggests that much physician training can be accomplished via medical simulation-based learning, simultaneously developing trainees’ proficiency and expertise while protecting patients from unnecessary risk and error.^17^ As Ziv et al. have eloquently noted, “The use of simulation when feasible conveys a critical educational and ethical message to all: patients are to be protected whenever possible and they are not commodities to be used as conveniences of training.”^17^ We believe that medical simulation should be used as a learning surrogate whenever possible.

## FOURTH ETHICAL CONCERN: APOLOGY AFTER AN ERROR

### Is error at all ameliorated by apology?

Once it becomes clear that ethical behavior still requires one to practice despite error, apology becomes an integral part of practice. Apology acknowledges the frailty of the human condition. It brings the physician to the level of the patient or family. Even if the patient has died, the family may take comfort from an apology. A sincere, empathic apology for causing error shows humanity and is integral to the commitment to practice of medicine. Concealing information on medical error may mar patient–doctor trust, alter a patient’s ability to make judicious decisions, and may ultimately lead to litigation.^18^

## CONCLUSION

Physicians, who long ago bound themselves by the spirit of the Hippocratic oath vowing first to do no harm, must continue to seek methods to identify and prevent error as well as remedy its effects.^19^ As Maimonides wrote in the 12th century, “May the love for my art actuate me at all times … today he can discover his errors of yesterday and tomorrow he can obtain a new light on what he thinks himself sure of today.”^20^ Physicians have a primary responsibility to the patient standing before them. As fallible physicians in the practice of medicine, we must continue to pursue our mission to serve and heal the sick to the best of our abilities.

## Abbreviations

IOM: Institute of Medicine
US: United States
